# Ets-1 is a transcriptional mediator of oncogenic nitric oxide signaling in estrogen receptor-negative breast cancer

**DOI:** 10.1186/bcr3319

**Published:** 2012-09-12

**Authors:** Christopher H Switzer, Robert Y-S Cheng, Lisa A Ridnour, Sharon A Glynn, Stefan Ambs, David A Wink

**Affiliations:** 1 Radiation Biology Branch, National Cancer Institute, NIH, 10 Center Drive, Bethesda, Maryland 20892 USA; 2 Laboratory of Human Carcinogenesis, National Cancer Institute, NIH, 37 Convent Drive, Bethesda, Maryland 20892 USA; 3 Prostate Cancer Institute, National University of Ireland Galway, Galway, Ireland

## Abstract

**Introduction:**

The Ets-1 transcription factor is a candidate breast cancer oncogene that regulates the expression of genes involved in tumor progression and metastasis. Ets-1 signaling has also been linked to the development of a basal-like breast cancer phenotype. We recently described a nitric oxide (NO)-induced gene signature that is associated with poor disease outcome in estrogen receptor-negative (ER-) breast cancer and contains both stem cell-like and basal-like components. Thus, we examined the role of Ets-1 in NO signaling and NO-induced phenotypes in ER- human breast cancer cells.

**Methods:**

Promoter region analyses were performed on genes upregulated in inducible nitric oxide synthase (NOS2) high expressing tumors for Ets-binding sites. *In vitro *mechanisms were examined in human basal-like breast cancer cells lines. NO signaling effects were studied using either forced NOS2 expression or the use of a chemical NO-donor, diethlylenetriamine NONOate (DETANO).

**Results:**

Promoter region analysis of genes that are up-regulated in human ER-negative breast tumors with high NOS2 expression revealed that the Ets-binding sequence is the only common promoter element present in all of these genes, indicating that Ets-1 is the key transcriptional factor down-stream of oncogenic NOS2-signaling. Accordingly, both forced NOS2 over-expression and exposure to NO-donors resulted in significant Ets-1 transcriptional activation in ER- breast cancer cells. Functional studies showed that NO activated Ets-1 transcriptional activity *via *a Ras/MEK/ERK signaling pathway by a mechanism that involved Ras S-nitrosylation. RNA knock-down of Ets-1 suppressed NO-induced expression of selected basal-like breast cancer markers such as P-cadherin, S100A8, IL-8 and αβ-crystallin. Additionally, Ets-1 knock-down reduced NO-mediated cellular proliferation, matrix metalloproteinase and cathepsin B activities, as well as matrigel invasion.

**Conclusions:**

These data show that Ets-1 is a key transcriptional mediator of oncogenic NO signaling that promotes the development of an aggressive disease phenotype in ER- breast cancer in an Ets-1 and Ras-dependent manner, providing novel clues of how NOS2 expression in human breast tumors is functionally linked to poor patient survival.

## Introduction

Inducible nitric oxide synthase (NOS2) is a pro-inflammatory enzyme generally with a key function in the innate immune response [1]. However, NOS2 expression is up-regulated and associated with poor outcome in many human cancers, such as melanoma, glioma and colon cancer [2-4]. Recently, we reported that high NOS2 expression is a predictor of poor patient outcome in estrogen receptor-negative (ER-) breast cancer and is functionally linked to the development of a basal-like breast cancer phenotype [5]. Basal-like tumors commonly present as the triple-negative disease, which limits the therapeutic options for the affected patients [6,7]. Nitric oxide (NO) signaling has various oncogenic effects in cancer cells [8-11]. For example, NO activates signaling through epidermanl growth factor receptor (EGFR), PI3K/Akt, HIF-1, and Src [5,12-15]. Together, these observations indicate that NOS2 expression may have deleterious effects in the progression of certain human cancers including ER- breast cancer. However, the molecular mechanisms by which NOS2 and NO signaling exerts an aggressive phenotype has yet to be fully determined.

Ets-1 is an oncogenic transcription factor involved in the progression of breast cancer [16-21]. Furthermore, tumor Ets-1 expression is linked to basal-like tumors and poor disease survival [19,22,23]. While Ets-1 is overexpressed in many tumors, its transcriptional activity is regulated at the phosphorylation level by extracellular signal-regulated protein kinases 1 and 2 (ERK1/2) [24-26]. Ets-1 regulates numerous genes involved in proliferation, angiogenesis, and metastasis [27]. For example, Ets-1 activity upregulates vascular endothelial growth factor (VEGF) [28] and matrix metalloproteinases (MMP) [29]. Thus, Ets-1 is a transcription factor that can promote an aggressive cancer cell phenotype.

Because both NOS2 and Ets-1 expression have oncogenic properties that advance the ER- disease, we investigated the functional relationship between them. This approach revealed that an Ets-binding sequence (EBS) is the only promoter element common to all genes in a previously described NOS2 expression signature for ER- breast tumors [5]. Furthermore, overexpression of NOS2 and experimental exposure to NO resulted in Ets-1 (threonine 38) phosphorylation and increased transcriptional activity in ER- breast cancer cell lines. Further analysis showed that NO activated Ets-1 *via *a Ras/mitogen-activated protein kinase (MEK)/ERK signaling axis by a mechanism that involved Ras S-nitrosylation (SNO). Finally, siRNA knock-down of Ets-1 also decreased NO-induced phenotypes of disease progression. Together, these data provide novel evidence that NO signaling promotes an aggressive breast cancer phenotype by activating the oncogenic Ets-1 transcription factor.

## Materials and methods

### Cell culture and reagents

Human breast adenocarcinoma cell lines MDA-MB-231, MDA-MB-468 and SKBR3 (American Type Culture Collection (ATCC), Manassas, VA, USA) were cultured in RPMI medium (Invitrogen, Carlsbad, CA, USA) containing 10% fetal bovine serum (Atlanta Biologics, Norcross, GA, USA) and 100 IU penicillin and 100 µg/ml streptomycin (Invitrogen). Cells were cultured at 37°C in 5% CO_2 _and passaged two to three times per week and were authenticated by short tandem repeat profiling within the past six months (ATCC). Aminoguanidine (AG) and L-arginine (L-Arg) were purchased from Sigma-Aldrich (St. Louis, MO, USA). Farnesylthiosalicylic acid (FTS) and PD 184161 were purchased from Cayman Chemical (Ann Arbor, MI, USA). Gö6976 was purchased from EMD Chemicals (Billerica, MA, USA). Recombinant human epidermal growth factor (EGF) was purchased from R&D Systems (Minneapolis, MN, USA). Antibodies to αβ-crystalin, actin, Ets-1 and NOS2 were from Santa Cruz Biotechnology (Santa Cruz, CA, USA). Antibodies to phospho-ERK1/2 (thr 202/tyr 204), ERK1/2 and phospho-MEK1/2 (ser 217/221) were from Cell Signaling (Danvers, MA, USA). Anti-Ras was from Thermo Scientific (Waltham, MA, USA) and anti-phospho-Ets-1 (thr 38) was purchased from Invitrogen. DETANO was generously provided by Dr. Larry Keefer (National Cancer Institute, Frederick, MD, USA). DETANO stock solutions were made in 10 mM NaOH and concentrations were determined by absorbance at 250 nm (ε = 8000 M^-1^·cm^-1^) prior to every use.

### Genomic sequence analyses

The promoter sequence for each gene listed in Table 3 of Glynn *et al*. [5] was extracted using ElDorado (version 12-2010) software and analyses were performed using the RegionMiner (Release 4.2) software. Both software packages are part of the commercially available Genomatix Software Suite (V2.1) (Genomatix Software, Inc, Ann Arbor, MI, USA).

### NOS2 expression

Cells were transfected with 4 µg pCMV6-XL4 (empty vector) or pCMV6-XL4-human NOS2 (NM_000625) (OriGene Technologies, Rockville, MD, USA) by electroporation using the Amaxa Nucleofector kit V (Lonza, Walkersville, MD, USA) and then grown for 48 hours under normal conditions before further treatment or analysis.

### Western blotting

Western blotting was performed by standard procedures. Cells were lysed on ice with cold lysis buffer (Tris-HCl pH 8.0 (50 mM), NaCl (150 mM), NP-40 (1%), ethylenediaminetetraacetic acid (EDTA, 1 mM), NaF (50 mM), Na_3_VO_4 _(10 mM), phenylmethylsulfonyl fluoride (PMSF, 1 mM) and protease inhibitor cocktail (EMD Chemicals)). Images were recorded on a FluoroChem SP system using AlphaEase FC software (Alpha Innotech, San Leandro, CA, USA).

### Ets-luciferase assays

Ets-1 transcriptional activity was performed by transiently transfecting cells with 750 ng of Ets-luciferase reporter plasmid expressing firefly luciferase (Panomics, Santa Clara, CA, USA) and 250 ng pGL4.70 plasmid expressing renella luciferase (Promega, Madison, WI, USA) using Lipofectamine LTX reagent for six hours at 37°C. After transfection, cell culture media was replaced with serum-free (D)MEM containing EGF (20 ng/mL), DETANO and inhibitors. Cells were incubated for 18 hours and luciferase activity was measured using the Dual-luciferase assay kit (Promega). Relative luminescent units (RLU) were measured using a Glomax 96-well plate luminometer (Promega) and data were normalized to fold change from untreated control cells. Data represent mean normalized RLU ± standard deviation (SD).

### Ras activation and S-nitrosylation

Relative Ras activation was determined using the Ras binding domain (RBD)-pull-down assay kit (Thermo Scientific). Briefly, cell lysate was incubated with RBD-agarose beads. Immunoprecipitated active Ras was eluted by boiling in 4X-lithium dodecyl sulfate (LDS) sample buffer. Active Ras and total cellular Ras were measured by western blot. Activation of Ras is shown as mean fold increase compared to untreated cells ± SD. Ras was immunoprecipitated using Protein G-Dynabeads (Invitrogen) conjugated with monoclonal mouse anti-Ras and assayed with the S-Nitrosylated Protein Detection Kit (Cayman Chemical) as instructed by the manufacturer. Procedures were performed under low ambient light to diminish Ras-SNO decomposition.

### Ets-1 knock-down

Cells (1 × 10^6^/100 µL) were transfected with 400 nM total siRNA by electroporation using the Amaxa Nucleofector Kit V. Cells were grown in RPMI + 10% FBS for 48 hours before further treatment or analysis. Human Ets-1 siGENOME SMARTpool (Thermo Scientific) oligonucleotide sequences: 5'-GAUAAAUCCUGUCAGUCUU-3'; 5'-GGACCGUGCUGACCUCAAU-3'; 5'-GGAAUUACUCACUGAUAAA-3' and 5'-GCAUAGAGAGCUACGAUAG-3'. Control siGENOME non-targeting siRNA pool (Thermo Scientific) sequences: 5'-UAGCGACUAAACACAUCAA-3'; 5'-UAAGGCUAUGAAGAGAUAC-3'; 5'-AUGUAUUGGCCUGUAUUAG-3' and 5'-AUGAACGUGAAUUGCUCAA-3'. Ets-1 knock-down was verified at the protein level by western blot.

### Proliferation assay

Cells were treated with or without 0.5 mM DETANO in serum-free RPMI containing 20 µM bromodeoxyuridine (BrDU) for 24 hours. Using the BrDU ELISA kit (Cell Signaling), cells were then fixed, washed and BrDU incorporation was determined by incubating mouse anti-BrDU followed by anti-mouse-horseradish peroxidase (HRP) secondary. Absorbance data are normalized to fold-increase compared to untreated controls and are shown as mean fold change ± SD.

### Cathepsin B activity

Cathepsin B Activity Fluorometric Assay Kit (Abcam, Cambridge, MA, USA) was used as instructed. Briefly, treated cells were lysed and samples were incubated with substrate Acetyl-arginine-arginine-amino-4-trifluoromethyl coumarin (Ac-RR-AFC). Released AFC was measured by fluorescence (400 nm excitation/505 nm emission). Data are normalized to fold change compared to untreated control cells and are shown as mean ± SD.

### MMP expression

MMP isoform expression was measured by spot-ELISA (R&D Systems) as instructed by the manufacturer. Briefly, conditioned medium was diluted and incubated in wells containing absorbed MMP antibodies. After washing, HRP-secondary antibody was applied and resulting spots were imaged by chemiluminescence as described above.

### MMP activity

Total MMP activity was measured by the MCa assay as previously described [30]. Briefly, conditioned medium was incubated with 10 µM MCa peptide [7-methoxycoumarin-4-acetyl-Pro-Leu-Gly-Leu-β-(2,4dinitrophenylamino)Ala-Ala-Arg-NH2] (Sigma-Aldrich). Fluorescence intensity was measured (328 nm excitation/392 nm emission) and normalized to total cellular protein. Data are represented as mean RFU per microgram protein ± SD.

### Cellular invasion

Cellular invasion assays were performed as previously described [5]. Briefly, MDA-MB-231 cells were seeded into the top chamber of transwell plates with 8 mm pores with a thin film of matrigel (BD Biosciences, San Jose, CA, USA) in serum-free RPMI containing the indicated concentration of DETANO and allowed to invade towards RPMI containing 5% FBS for 24 hours. Data represent mean number of invading cells ± SD.

### Statistical analyses

Data analyses were performed using Prism 4 software (GraphPad Software, Inc., La Jolla, CA, USA). Statistical significance was calculated using one-way analysis of variance (ANOVA) analyses with Dunnett's post-test or unpaired t-test. Significance was determined with *P *values less than 0.05 or 0.01 as stated in the figure legends.

## Results

### NOS2 signals through Ets-1 in human ER- breast tumors

Recently, we reported that NOS2 expression is significantly associated with poor survival in ER- breast cancer and that high NOS2 expression is associated with a distinct gene expression profile similar to the basal-like phenotype [5]. Further analysis of the gene signature revealed that the Ets-binding site (EBS) is the only promoter element common to all 46 up-regulated genes [see Additional file 1: Table S1]. To further examine the enrichment of EBS-regulated genes in high NOS2 expressing ER- tumors, bulk tumor tissue was also analyzed using the Gene Set Analysis against the TRANSFAC database. A significant enrichment of genes with EBS was found among the genes that were up-regulated in the NOS2 high tumors, confirming that NOS2 and Ets-regulated genes are correlated in ER- breast tumors. Thus, we examined the role of NOS2 activity and NO signaling in the activation of the Ets-1 transcription factor in human ER- breast cancer cell lines.

### NOS2 and NO increases Ets-1 transcriptional activity

**Figure 1:**
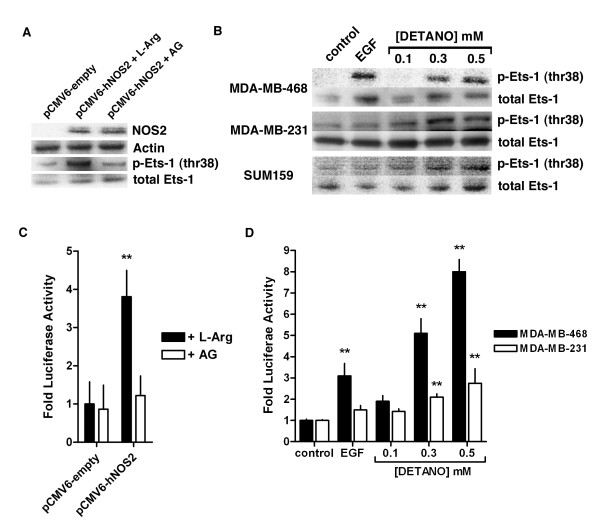
**Ets-1 transcriptional activity in response to NOS2 expression and NO signaling**. **(a) **Western blot of NOS2 and Ets-1 (thr 38) phosphorylation in MDA-MB-468 cells transfected with control plasmid and NOS2 expression plasmid in the presence of NOS2 substrate (L-Arg) or inhibitor (AG). **(b) **Western blot of phospho-Ets-1 (thr 38) compared to total Ets-1 in serum-starved cells exposed to either EGF (10 ng/ml) or DETANO. **(c) **Ets-luciferase activity in MDA-MB-468 cells transfected with either control or NOS2 expression plasmid and cultured in the presence of L-Arg or AG. Data represent mean fold luciferase activity compared to control plasmid incubated with L-Arg. **(d) **Ets-luciferase activity in serum-starved cells treated with either EGF or DETANO. Data represent mean fold luciferase activity compared to untreated control. Significant luciferase activity (**P < 0.01) was determined by one-way ANOVA from at least three independent experiments. AG, aminoguanidine; ANOVA, analysis of variance; DETANO, diethlylenetriamine NONOate; EGF, epidermal growth factor; Ets-1, erythroblastosis virus E26 oncogene homolog 1; L-Arg, L-arginine; NOS2, nitric oxide synthase.

To evaluate Ets-1 activation by NO signaling, we examined the effect of forced NOS2 expression on Ets-1 (thr 38) phosphorylation in human basal-like cells. MDA-MB-468 cells, which do not express basal NOS2, were transfected with a human NOS2 expression plasmid and incubated with the NOS2 substrate L-Arg or the NOS2 inhibitor AG. NOS2 expression in the presence of L-Arg resulted in robust Ets-1 (thr 38) phosphorylation compared to cells transfected with empty vector control (Figure 1A). Ets-1 (thr 38) phosphorylation was markedly reduced in NOS2 expressing cells treated with AG.

Because NOS2 expression resulted in Ets-1 (thr 38) phosphorylation, we also examined the effect of NO signaling on Ets-1 activation in human ER- breast cancer cell lines treated with NO releasing compounds. Using the chemical NO-donor DETANO, the effect of NO on Ets-1 (thr 38) phosphorylation in MDA-MB-468, MDA-MB-231 and SUM159 cell lines was examined. The applied donor concentrations generate actual NO concentrations that are in the physiological nanomolar concentration range because of the slow release rate of NO from this donor [see Additional file 2: Figure S1] [31]. DETANO induced significant increases in Ets-1 (thr 38) phosphorylation in all three cell lines in a concentration-dependent manner as compared to untreated serum-starved controls (Figure 1B). The NO-donor at 0.5 mM induced a level of Ets-1 (thr 38) phosphorylation similar to the stimulation of MDA-MB-468 cells with EGF (10 ng/ml). EGF did not result in an increase of Ets-1 (thr 38) phosphorylation in MDA-MB-231 or SUM159 cell lines, which exhibit relatively low EGFR expression and EGF-induced tyrosine 1173 phosphorylation compared to MDA-MB-468 cells [see Additional file 3: Figure S2]. Additionally, similar results were observed in the ER-/HER2+ SKBR3 cell line [see Additional file 4: Figure S3]. Our data indicate that NOS2 phosphorylates Ets-1 *via *NO production and subsequent NO signaling.

To examine the effect of NOS2 expression on Ets-1 transcriptional activity, MDA-MB-468 cells were transfected with a NOS2 expression plasmid and then transiently transfected with an Ets-luciferase reporter plasmid. Cells were then incubated in serum-free media supplemented with L-Arg or AG. NOS2 expression resulted in a significant increase in luciferase reporter activity when incubated with L-Arg; however, this effect was not observed in the presence of the NOS2 inhibitor AG, indicating that NO release resulted in Ets-1 transcriptional activation (Figure 1C). To examine the effect of NO signaling on Ets-1 transcriptional activity, MDA-MB-468 and MDA-MB-231 cells were transiently transfected with an Ets-luciferase reporter plasmid and treated with EGF or DETANO in serum-free media. EGF caused a significant increase in luciferase activity compared to untreated controls in the MDA-MB-468 cells, but not in MDA-MB-231 cells, reminiscent of the Ets-1 (thr 38) phosphorylation findings for these cell lines (Figure 1D). DETANO caused a concentration-dependent increase in luciferase activity and the effect was most significant at 0.3 and 0.5 mM in both MDA-MB-468 and MDA-MB-231 cells. These data show that NOS2, *via *NO signaling, increases Ets-1 transcriptional activity in ER- breast cancer cells.

### NO activates Ets-1 *via *a Ras/MEK/ERK signaling pathway

**Figure 2:**
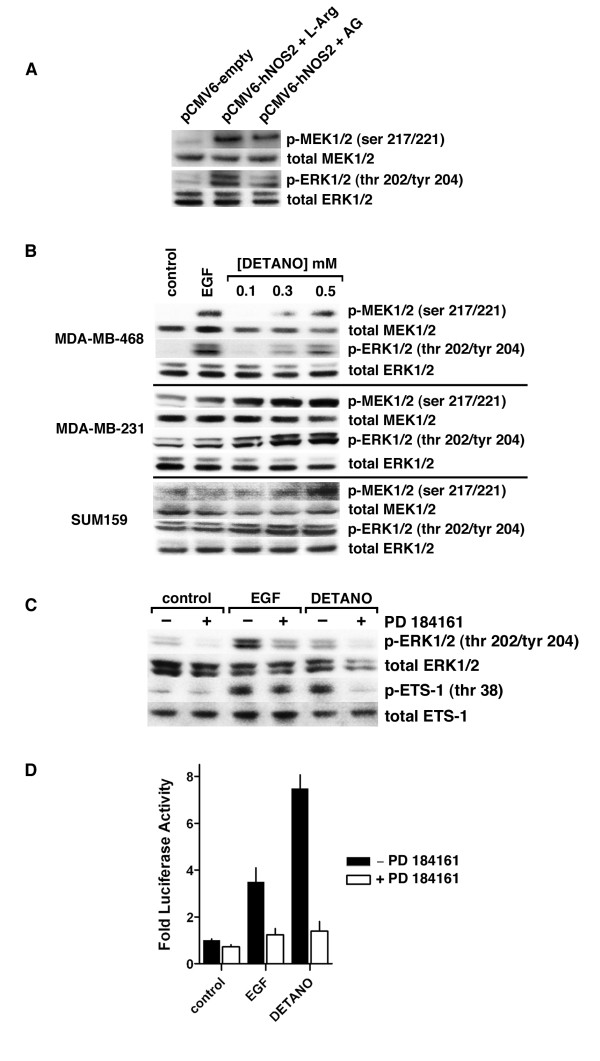
**NO activation of Ets-1 requires the MEK/ERK signaling pathway**. **(a) **Western blot of relative MEK1/2 (ser 217/221) and ERK1/2 (thr 202/tyr204) phosphorylation in MDA-MB-468 cells transfected with control or NOS2 expression plasmid and cultured with L-Arg or AG. **(b) **Western blot of relative MEK1/2 (ser 217/221) and ERK1/2 (thr 202/tyr204) phosphorylation in serum-starved cells exposed to EGF or DETANO. **(c) **Western blot of ERK1/2 (thr 202/tyr204) and Ets-1 (thr 38) phosphorylation in serum-starved MDA-MB-468 cells exposed to EGF (10 ng/ml) or DETANO (0.5 mM), with and without the MEK inhibitor PD 184161. **(d) **Ets-luciferase activity in serum-starved MDA-MB-468 cells exposed to conditions described in (c). AG, aminoguanidine; DETANO, diethlylenetriamine NONOate; EGF, epidermal growth factor; ERK, extracellular signal-regulated protein kinase; Ets-1, erythroblastosis virus E26 oncogene homolog 1; L-Arg, L-arginine; MEK, mitogen-activated protein kinase; NOS2, nitric oxide synthase.

Ets-1 is phosphorylated and activated by the MEK/ERK signaling pathway [26]. Therefore, the role of MEK/ERK signaling was examined in NO-induced Ets1 activation. Transfection of MDA-MB-468 cells with a NOS2 expression plasmid resulted in increased MEK1/2 (ser 217/221) and ERK1/2 (thr 202/tyr 204) phosphorylation compared to control cells and this effect was reduced in the presence of AG (Figure 2A). DETANO caused a concentration-dependent increase in both MEK1/2 (ser 217/221) and ERK1/2 (thr 202/tyr 204) phosphorylation in MDA-MB-231, MDA-MB-468 and SUM159 cells (Figure 2B). Similar results were obtained in SKBR3 cells [see Additional file 4: Figure S3]. Furthermore, the DETANO-mediated phosphorylation of ERK1/2 (thr 202/tyr 204) and p-Ets-1 (thr 38) was attenuated by the MEK inhibitor PD184161 in MDA-MB-468 cells (Figure 2C). Ets-luciferase activity in MDA-MB-468 cells treated with either EGF or 0.5 mM DETANO was significantly decreased in the presence of PD184161 compared to EGF or DETANO alone (Figure 2D). These data show that NO activates Ets-1 *via *the MEK/ERK signaling pathway.

**Figure 3:**
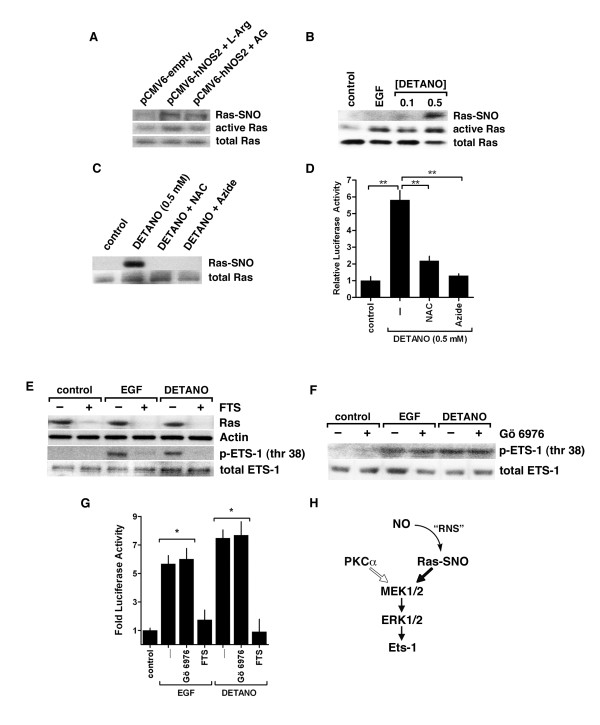
**Ets-1 activation by NO requires Ras signaling**. Western blot of S-nitrosylated, active and total Ras in serum-starved MDA-MB-468 cells **(a) **transfected with NOS2 expression plasmid and treated with L-Arg or AG and **(b) **treated with EGF or DETANO. **(c) **Western blot of Ras-SNO and total Ras from MDA-MB-468 cells exposed to DETANO (0.5 mM) alone or in combination with N-acetyl cysteine (NAC) or sodium azide. **(d) **Ets-luciferase activity in MDA-MB-468 cells exposed to conditions as above. Significance to DETANO was determined by one-way ANOVA (**P < 0.01). **(e) **Western blot of Ras and Ets-1 (thr 38) phosphorylation in serum-starved MDA-MB-468 cells exposed to EGF or DETANO (0.5 mM) in the presence or absence of FTS. **(f) **Western blot of Ets-1 (thr 38) phosphorylation in serum-starved MDA-MB-468 cells exposed to EGF or DETANO (0.5 mM) in the presence or absence of Gö 6976. **(g) **Ets-luciferase activity in serum-starved MDA-MB-468 cells exposed to EGF or DETANO (0.5 mM) in the presence or absence of FTS or Gö 6976. Significance compared to control was determined by one-way ANOVA (*P < 0.05). **(h) **Schematic representing the NO-sensitive Ras/MEK/ERK/Ets-1 signaling pathway. AG, aminoguanidine; ANOVA, analysis of variance; DETANO, diethlylenetriamine NONOate; EGF, epidermal growth factor; ERK, extracellular signal-regulated protein kinase; Ets-1, erythroblastosis virus E26 oncogene homolog 1; FTS, farnesylthiosalicylic acid; L-Arg, L-arginine; MEK, mitogen-activated protein kinase; NOS2, nitric oxide synthase.

Ras is a major activator of MEK/ERK signaling [32], therefore the role of Ras signaling in mediating NOS2 and NO-induced Ets-1 activation was examined. Wild type Ras expressing MDA-MB-468 cells were transfected as described above and the relative level of Ras activation was determined by the RBD pull-down assay and compared to total Ras expression. NOS2 expression in the presence of L-Arg resulted in Ras activation compared to control cells; however, the addition of AG reduced levels of active Ras (Figure 3A). Because NO activates Ras *via *SNO post-translational modification [33,34], Ras-SNO formation was measured by the biotin-switch assay [35]. Similar to Ras activation, NOS2 expression resulted in Ras-SNO, which was reduced in the presence of AG (Figure 3A). To examine the effect of NO on Ras activation and S-nitrosylation, MDA-MB-468 cells were treated with either EGF or DETANO for 24 hours. Ras activation was significantly increased by EGF and both concentrations of DETANO (0.1 and 0.5 mM) compared to serum-starved controls (Figure 3B). Densitometric analyses show that DETANO at 0.5 mM activated Ras comparable to EGF (*P *< 0.01), whereas 0.1 mM DETANO induced an activation that was significantly lower than EGF, albeit still statistically significant above control levels [see Additional file 5: Figure S4]. Ras-SNO formation was observed in MDA-MB-468 cells treated with 0.5 mM but not with 0.1 mM DETANO consistent with a nitrosative signaling profile of NO (Figure 3B) [36]. Ras-SNO was not observed in control or EGF stimulated cells. To further examine the role of Ras-SNO modification in the activation of Ets-1, MDA-MB-468 cells were treated with DETANO alone or in the presence of chemical inhibitors of S-nitrosation, N-acetyl cysteine (NAC) or sodium azide. Ras-SNO was detected in cells treated with DETANO; however, both NAC and azide blocked Ras-SNO formation (Figure 3C). Ets-luciferase activity was measured in MDA-MB-468 cells treated with DETANO alone and in combination with NAC or azide. DETANO resulted in increased luciferase activity compared to untreated controls and NAC and azide significantly reduced NO-mediated Ets-1 transcriptional activity (Figure 3D). These results suggest that activation of Ras and Ets-1 by 0.5 mM DETANO is mediated, at least in part, by Ras-SNO formation.

To examine the role of Ras in mediating the NO activation of the MEK/ERK/Ets-1 signaling pathway, MDA-MB-468 cells were treated with EGF or 0.5 mM DETANO with or without the Ras inhibitor FTS. FTS blocks Ras association with the cellular membrane and renders Ras protein susceptible to proteasomal degradation [37]. EGF and DETANO resulted in Ets-1 (thr 38) phosphorylation; however, this signaling effect was not observed in the presence of FTS (Figure 3E). Furthermore, FTS treatment resulted in decreased Ras protein levels, indicating that Ras signaling is critical for NO to increase Ets-1 (thr 38) phosphorylation. An alternative activator of MEK-1/2 signaling is protein kinase Cα (PKCα) [38-40]. To examine the role of PKCα on NO activation of MEK/ERK/Ets-1 signaling, cells were treated with EGF or 0.5 mM DETANO and with or without the PKCα inhibitor Gö 6976. The phosphorylation of Ets-1 by NO was not altered by Gö 6976 (Figure 3F), suggesting that NO activates Ets-1 *via *a PKCα-independent mechanism.

To examine the role of Ras and PKCα on NO-mediated Ets-1 transcriptional activity, MDA-MB-468 cells were transfected with an Ets-luciferase reporter plasmid and treated with 0.5 mM DETANO alone or in combination with either Gö 6976 or FTS. Consistent with the Ets-1 phosphorylation results, FTS blocked the effect of NO to increase Ets-1 transcriptional activity, while Gö 6976 had no effect on luciferase activity (Figure 3G). These data suggest that NO activates Ets-1 signaling and its transcriptional activity *via *a Ras/MEK/ERK signaling pathway and not *via *PKCα activation (Figure 3H).

### NO and Ets-1 contribute to an aggressive basal-like phenotype

**Figure 4:**
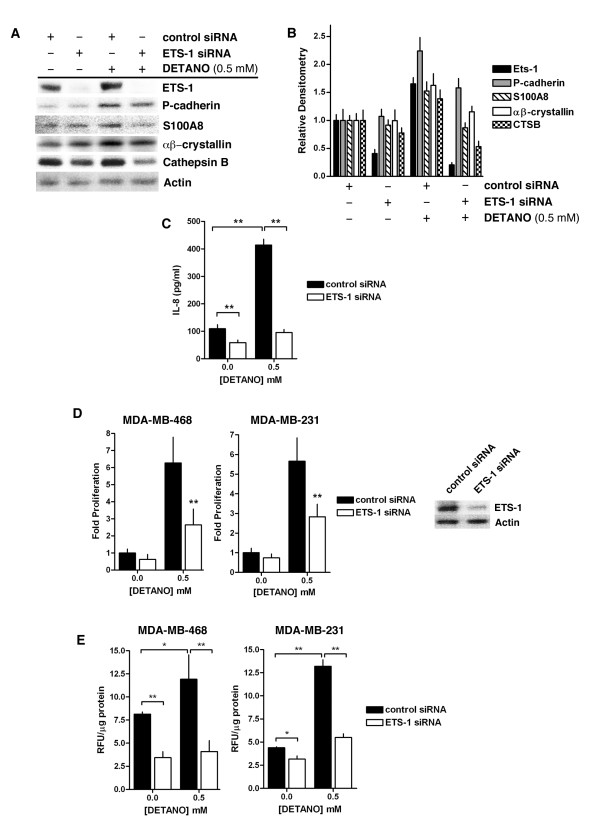
**Ets-1 mediates the NO-induced aggressive basal-like phenotype**. **(a) **Representative western blots of Ets-1, P-cadherin, S100A8, αβ-crystallin, cathepsin B (CTSB) and actin expression in MDA-MB-468 cells transfected with either control or Ets-1 siRNA and treated with DETANO. **(b) **Densitometic analyses of proteins described in (a) relative to untreated control plasmid cells. **(c) **IL-8 production from MDA-MB-468 cells transfected with either control or Ets-1 siRNA and treated with DETANO. Significance (**P < 0.01) was determined by t-test. **(d) **Proliferation of MDA-MB-468 cells transfected with control or Ets-1 siRNA and treated with DETANO in serum-free RPMI. Data represent the fold change compared to untreated, control siRNA cells. Significance compared to control siRNA transfected cells was determined by t-test (**P < 0.01). (Inset: Western blot of Ets-1 expression compared to actin in transfected MDA-MB-231 cells.) **(e**) CTSB activity in cells transfected with control or Ets-1 siRNA and treated with DETANO. Relative fluorescence units (RFU) were normalized to µg of total protein ± SD. Significance (*P < 0.05, **P < 0.01) was determined by t-test. DETANO, diethlylenetriamine NONOate; Ets-1, erythroblastosis virus E26 oncogene homolog 1; SD, standard deviation.

NOS2 expression is associated with a basal-like phenotype in ER- breast tumors and NO signaling results in increased expression of basal-like signature genes in ER- human breast cancer cell lines [5]. To examine the role of Ets-1 in mediating the expression of basal-like markers induced by NO signaling, MDA-MB-468 cells were transfected with either control or Ets-1-specific siRNA and exposed to DETANO. Western blotting showed that Ets-1 siRNA resulted in suppression of Ets-1 protein expression (Figure 4A). DETANO treatment resulted in increased expression of the basal-like markers P-cadherin, S100A8 and αβ-crystallin when compared to control siRNA treated cells (Figure 4A). Furthermore, the increase of P-cadherin, S100A8 and αβ-crystallin expression by DETANO was reduced in Ets-1 knocked-down cells (Figure 4A). Densitometric analyses of protein expression displayed in Figure 4A are shown in Figure 4B. In addition, IL-8 production was significantly increased by NO and significantly reduced in Ets-1 siRNA transfected MDA-MB-468 cells (Figure 4C). Similarly, the increased cellular proliferation induced by DETANO treatment was significantly reduced in Ets-1 siRNA transfected MDA-MB-468 and MDA-MB-231 cells (Figure 4D). These data show that Ets-1 mediates the expression of the basal-like breast cancer signature genes induced by oncogenic NO signaling.

Ets-1 regulates the expression of various proteases that are critical to matrix reorganization and cancer cell invasion [41]. Therefore, the role of NO/Ets-1 signaling on cathepsin B (CTSB) was examined. CTSB expression and activity was measured in extracts from cells transfected with Ets-1 siRNA and treated with or without 0.5 mM DETANO and compared to cells transfected with control siRNA. CTSB expression was only modestly increased in DETANO-treated control cells but was markedly reduced in cells transfected with Ets-1 siRNA (Figure 4A and 4B). In contrast to the CTSB expression levels, CTSB activity significantly increased in DETANO-treated cells when compared to untreated cells (Figure 4D). However, CTSB activity was significantly reduced in cells transfected with Ets-1 siRNA compared to control siRNA in both DETANO-treated and untreated conditions (Figure 4E). These results show that NO increases CTSB expression and activity *via *Ets-1 signaling.

**Figure 5:**
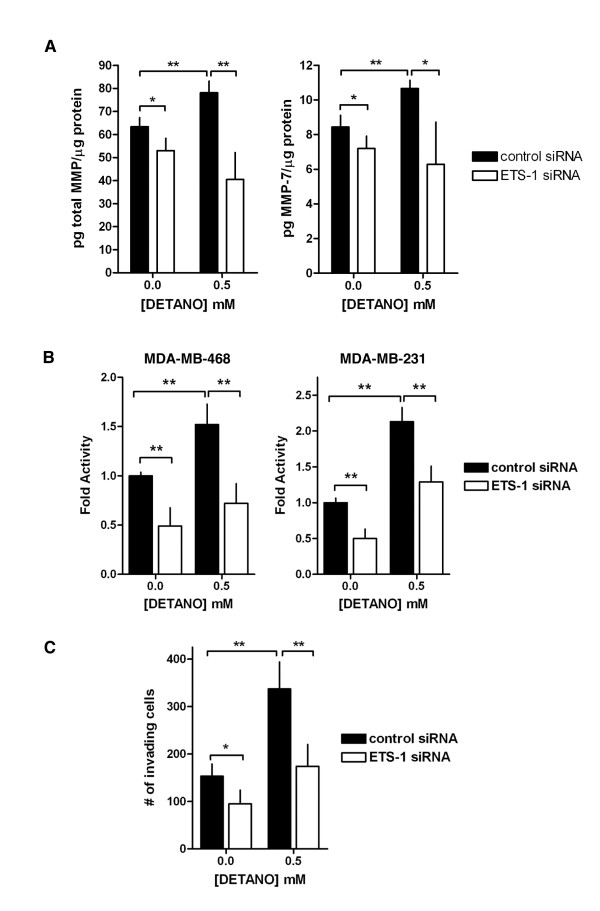
**Ets-1 mediates NO-induced MMP activity and cellular invasion**. Total MMP and MMP-7 expression **(a) **in conditioned media from MDA-MB-468 cells transfected with control or Ets-1 siRNA and treated with DETANO. MMP expression is normalized to total cellular protein. **(b) **Total MMP activity in serum-starved cells transfected with control or Ets-1 siRNA and treated with DETANO. Activity is shown as fold change compared to untreated, control cells. **(c) **MDA-MB-231 cell invasion in response to control or Ets-1 siRNA transfection and DETANO exposure. Significance for expression, activity and invasion data were determined by t-test (*P < 0.05, **P < 0.01). DETANO, diethlylenetriamine NONOate; Ets-1, erythroblastosis virus E26 oncogene homolog 1; MMP, matrix metalloproteinase.

Ets-1 regulates the expression of many proteases of the MMP family [17,42,43], which accelerate tumor cell invasion and metastasis [44]. To examine the role of Ets-1 in mediating NO-induced MMP expression, conditioned media were assayed for total MMP expression using a mosaic MMP spot-ELISA which measures MMP-1, -2, -3, -7, -8, -9 and -13. Total MMP (that is, the sum of the MMPs measured) was significantly decreased in cells transfected with Ets-1 siRNA (Figure 5A). DETANO treatment resulted in a moderate albeit significant increase of total MMP and this effect was suppressed in Ets-1 siRNA cells (Figure 5A). The most abundant MMP measured in conditioned media was MMP-7 (matrilysin) and both NO and Ets-1 knock-down had effects on MMP-7 expression similar to those of total MMP expression (Figure 5A). Total MMP activity was also measured from conditioned media using the Mca assay. Cells transfected with Ets-1 siRNA exhibited a significant reduction in MMP activity compared to cells transfected with control siRNA (Figure 5B). Control cells treated with 0.5 mM DETANO had a significant increase in MMP activity and this effect was significantly reduced in Ets-1 knock-down cells (Figure 5B). The role of Ets-1 in mediating NO-induced MDA-MB-231 invasion was also measured using the matrigel invasion assay. Similar to MMP activity, cellular invasion was reduced in Ets-1 siRNA transfected cells compared to control siRNA transfected cells (Figure 5C). Control cells treated with 0.5 mM DETANO exhibited increased invasion compared to cells not exposed to DETANO and this effect was significantly reduced in Ets-1 knock-down cells (Figure 5C). These data indicate that Ets-1 has a critical role in the NO-induced cellular proliferation, invasion and expression of basal-like markers in ER- breast cancer cells.

## Discussion

Our study made the novel observation that the oncogenic transcription factor Ets-1 is a critical mediator of NOS2 and NO-induced signaling in breast cancer and thus, this study provides a molecular mechanism that at least partly explains the oncogenic effects of NO in ER- breast cancer. Moreover, the robust association between NOS2 expression and up-regulation of genes with EBS transcriptional activation sites in microdissected and bulk tumor epithelia indicates that Ets-1 is a significant *in vivo *mediator of NOS2 signaling in human ER- breast tumors. NOS2 expression in ER- breast tumors is associated with poor patient outcomes and a basal-like phenotype [5], linking NO signaling to this poor outcome and highly metastatic phenotype [6,45]. NO activation of Ets-1 resulted in the cellular expression of basal-like markers (P-cadherin, S100A8, IL-8 and αβ-crystallin) [46-48] as well as molecules associated with the metastatic process (CTSB and MMP-7) [49,50] indicating that this signaling mechanism contributes to the observed clinical features of aggressive ER- breast cancers that overexpress NOS2. In addition to the Ras/MEK/ERK/Ets-1 signaling pathway elucidated here, NOS2 and NO activate multiple oncogenic signaling pathways such as EGFR, PI3K/Akt, c-Myc, HIF-1a, NF-kB and Src [8]. Furthermore, S100A8 and MMPs are potential targets of SNO highlighting the multifaceted effects of NO signaling in cancer cell biology. Therefore, the activation of Ras/Ets-1 is a contributing signaling axis induced by oncogenic levels of NO [8,51]. These observations strongly point to NOS2 as a potential comprehensive driver of aggressive metastatic tumors and further suggest that NOS2 inhibition or blunting of NO/SNO signaling is a potential therapeutic target for basal-like breast tumors. This is of significant clinical impact as basal-like tumors commonly express the triple-negative phenotype and, therefore, currently lack therapeutic targets [6,7].

The data shown here indicate that Ras activation by NO has signaling effects in human breast cancer and this signaling mechanism may represent a major target of NO signaling in cancer biology. While mutated and constitutively active Ras is often observed in human malignancy, breast tumors harboring Ras mutations are rare, accounting for >5% of all breast tumors [52,53]. Wild-type Ras-SNO modification and activation has been characterized; however, the resulting signaling effects in human cancer have not been thoroughly investigated. The involvement of Ras SNO described here in ER- breast cancer cells is consistent with previous reports in T lymphocytes and lung tumors [54,55]. Ras activation by NO in breast cancer cells has been described to proceed in a cGMP-independnet mechanism and our data showing NO-mediated SNO of Ras is consistent with this previous report [56]. Our finding that NO activation of Ras, *via *SNO results in Ets-1 activation suggests that other Ras-mediated pathways may also activated by NO in human cancer.

We propose that the NO/Ets-1 signaling axis first described here may promote disease progression in other tumors that overexpress NOS2, such as glioma and melanoma [2,57], and tumors with impaired SNO metabolism, such as lung and hepatocellular carcinoma [55,58]. Ets-1 has also been linked to melanoma and lung tumor metastasis [59,60]. Furthermore, our data showing that NO results in a MEK/ERK/Ets-1 signaling cascade in ER-/HER2+ SKBR3 cells [see Additional file 4: Figure S3] suggest that high NOS2 expression and NO signaling may induce proliferative and aggressive phenotypes in HER2+ breast cancer. Together, these data further strengthen the proposed linkage between NO and Ets-1 signaling and suggest that their interaction is a major promoter of tumor metastasis and requires further investigation.

## Conclusions

In summary, NO signaling results in the activation of the oncogenic transcription factor Ets-1, which is critical for the basal-like breast cancer phenotype associated with tumor NOS2 expression. This effect of NO is mediated by Ras-SNO modification and subsequent MEK/ERK signaling to phosphorylate Ets-1 (thr 38). Activation of Ets-1 by NO resulted in the increased expression of the basal-like markers P-cadherin, S100A8, IL-8 and αβ-crystallin, which mechanistically links two prognostic markers of poor basal-like patient survival [5,23]. Furthermore, NO activation of Ets-1 resulted in increased expression and activity of proteases critical for tumor metastasis, MMPs and CTSB, and resulted in increased cancer cell invasion and proliferation. These data imply a molecular mechanism that elucidates the aggressive basal-like phenotype induced by NOS2 and NO signaling and provides a potential therapeutic target for triple negative/basal-like breast cancer.

## Abbreviations

AG: aminoguanidine; cGMP: cyclic guanosine monophosphate; CTSB: cathepsin B; DETANO: diethlylenetriamine NONOate; (D)MEM: (Dulbecco's) modified Eagle's medium; EBS: Ets-binding sequence; EGF: epidermal growth factor; EGFR: epidermal growth factor receptor; ELISA: enzyme-linked immunosorbent assay; ER-: estrogen receptor-alpha negative; ERK: extracellular signal-regulated kinases; Ets-1: erythroblastosis virus E26 oncogene homolog 1; FTS: farnesylthiosalicylic acid; HRP: horseradish peroxidase; IL-8: interleukin-8; L-Arg: L-arginine; MEK: mitogen-activated protein kinase; MMP: matrix metalloproteinase; NAC: N-acetyl cysteine; NO: nitric oxide; NOS2: inducible nitric oxide synthase; PI3K: phosphatidylinositol 3-kinase; PLCα: protein kinase Cα; RBD: Ras-binding domain; RLU: relative luminescent units; SD: standard deviation; siRNA: small interfering RNA; SNO: S-nitrosylation; VEGF: vascular endothelial growth factor.

## Competing interests

The authors declare that they have no competing interests.

## Authors' contributions

CS conceived and designed experiments, acquired and analyzed data, and wrote and edited the manuscript. RC performed genomic analyses. LR measured steady state NO levels and assisted with MMP experiments. SG performed experiments and edited the manuscript. SA edited and rewrote the manuscript. DW helped with data analysis and funding for this study. All authors read and approved the final manuscript for publication.

## Supplementary Material

Additional file 1 **Table S1. Ets-binding sites in NOS2-associated gene signature**. An Excel table listing the Ets-binding sites within the ER- NOS2 gene signature. Click here for file

Additional file 2 **Figure S1. Steady-state NO concentrations released from DETANO**. A pdf file showing the concentration of NO, as measured by chemiluminescence, versus the concentration of DETANO over 24-hours. Click here for file

Additional file 3 **Figure S2. Response to EGF stimulation in ER- cell lines used in this study**. A pdf file showing a western blot comparing relative phospho-(tyr1173) and total EGFR expression in EGF-treated MDA-MB-468, MDA-MB-231, SUM159 and SKBR3 cells. Click here for file

Additional file 4 **Figure S3. NO activation of Ets-1 in ER-/HER2+ SKBR3 cells**. A pdf file showing a western blot of relative Ets-1 (thr 38), MEK1/2 (ser 217/221) and ERK1/2 (thr 202/tyr 204) phosphorylation in serum starved SKBR3 cells exposed to either EGF (10 ng/ml) or DETANO. Click here for file

Additional file 5 **Figure S4. Relative Ras activation of MDA-MB-468 cells**. A pdf file showing Ras activation as calculated from densitometric analyses of active Ras normalized to total Ras. Activity is shown as mean fold compared to control. Significance (*P < 0.05, **P < 0.01) was determined by t-test. Click here for file
