# Supplementary material for: Ets-1 is a transcriptional mediator of oncogenic nitric oxide signaling in estrogen receptor-negative breast cancer
Source: Breast Cancer Res. 2012 Sep 12;14(5):R125. doi: 10.1186/bcr3319 (PMC4053102; doi:10.1186/bcr3319)
Supplement: Additional file 2 — Figure S1. Steady-state NO concentrations released from DETANO. A pdf file showing the concentration of NO, as measured by chemiluminescence, versus the concentration of DETANO over 24-hours. [file bcr3319-S2.PDF]

Additional file 2: Figure S1.

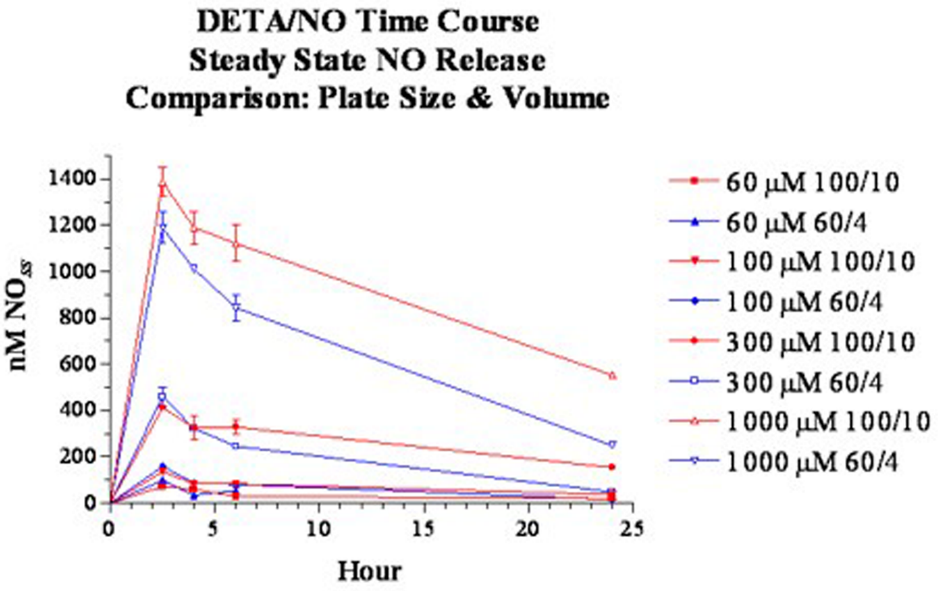

Steady-state NO concentrations released from DETANO.

DETANO was incubated for the indicated times and concentrations in RPMI and NO concentrations were measured by chemiluminescence. The concentration of DETANO (µM), diameter of the dish (mm) and volume of media (mL) are indicated in the legend.
