# Supplementary material for: Ets-1 is a transcriptional mediator of oncogenic nitric oxide signaling in estrogen receptor-negative breast cancer
Source: Breast Cancer Res. 2012 Sep 12;14(5):R125. doi: 10.1186/bcr3319 (PMC4053102; doi:10.1186/bcr3319)
Supplement: Additional file 3 — Figure S2. Response to EGF stimulation in ER- cell lines used in this study. A pdf file showing a western blot comparing relative phospho-(tyr1173) and total EGFR expression in EGF-treated MDA-MB-468, MDA-MB-231, SUM159 and SKBR3 cells. [file bcr3319-S3.PDF]

Additional file 3: Figure S2.

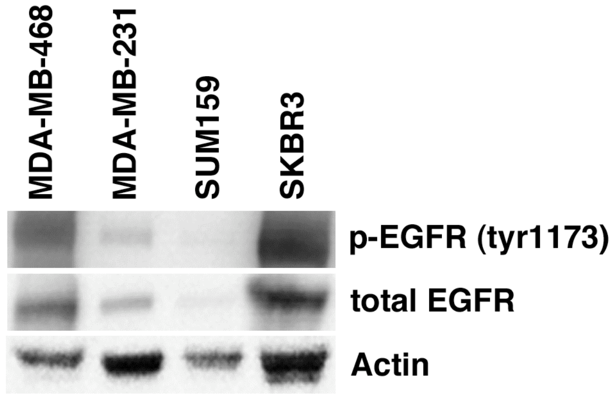

Response to EGF stimulation in ER- cell lines used in this study.
Western blot comparing relative phospho-(tyr1173) and total EGFR expression in EGF-treated MDA-MB-468, MDA-MB-231, SUM159 and SKBR3 cells.
