# Supplementary material for: Ets-1 is a transcriptional mediator of oncogenic nitric oxide signaling in estrogen receptor-negative breast cancer
Source: Breast Cancer Res. 2012 Sep 12;14(5):R125. doi: 10.1186/bcr3319 (PMC4053102; doi:10.1186/bcr3319)
Supplement: Additional file 4 — Figure S3. NO activation of Ets-1 in ER-/HER2+ SKBR3 cells. A pdf file showing a western blot of relative Ets-1 (thr 38), MEK1/2 (ser 217/221) and ERK1/2 (thr 202/tyr 204) phosphorylation in serum starved SKBR3 cells exposed to either EGF (10 ng/ml) or DETANO. [file bcr3319-S4.PDF]

Additional file 4: Figure S3.

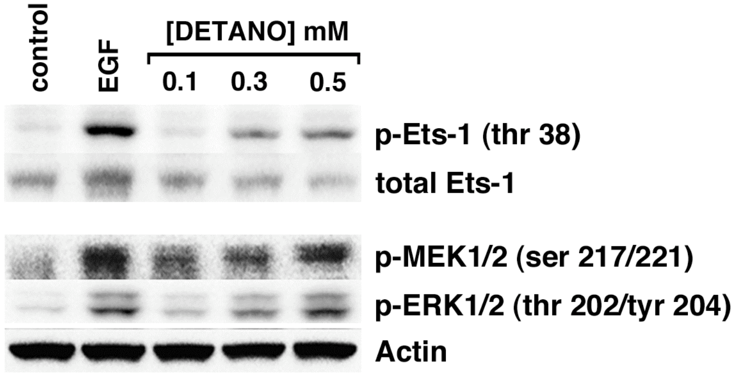

NO activation of Ets-1 in ER-/HER2+ SKBR3 cells.
Western blot of relative Ets-1 (thr 38), MEK1/2 (ser 217/221) and ERK1/2 (thr 202/tyr 204) phosphorylation in serum starved SKBR3 cells exposed to either EGF (10 ng/ml) or DETANO.
