# Supplementary material for: Ets-1 is a transcriptional mediator of oncogenic nitric oxide signaling in estrogen receptor-negative breast cancer
Source: Breast Cancer Res. 2012 Sep 12;14(5):R125. doi: 10.1186/bcr3319 (PMC4053102; doi:10.1186/bcr3319)
Supplement: Additional file 5 — Figure S4. Relative Ras activation of MDA-MB-468 cells. A pdf file showing Ras activation as calculated from densitometric analyses of active Ras normalized to total Ras. Activity is shown as mean fold compared to control. Significance (*P < 0.05, **P < 0.01) was determined by t-test. [file bcr3319-S5.PDF]

Additional file 5: Figure S4.

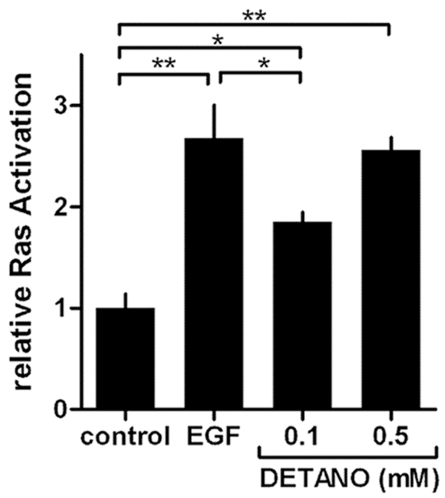

Relative Ras activation of MDA-MB-468 cells.

Ras activation calculated from densitometric analyses of active Ras normalized to total Ras. Activity is shown as mean fold compared to control. Significance (*$P < 0.05$, **$P < 0.01$) was determined by t-test.
